# ICENET: An Information Centric Protocol for Big Data Wireless Sensor Networks

**DOI:** 10.3390/s19040930

**Published:** 2019-02-22

**Authors:** Rosana Lachowski, Marcelo E. Pellenz, Edgard Jamhour, Manoel C. Penna, Glauber Brante, Guilherme Moritz, Richard D. Souza

**Affiliations:** 1 PPGIa, Pontifical Catholic University of Parana, Parana 80215-901, Curitiba, Brazil; rosana@ppgia.pucpr.br (R.L.); edgard.jamhour@pucpr.br (E.J.); penna@ppgia.pucpr.br (M.C.P.); 2 CPGEI, Federal University of Technology, Parana 81531-990, Curitiba, Brazil; gbrante@utfpr.edu.br (G.B.); moritz@utfpr.edu.br (G.M.); 3 UFSC, Federal University of Santa Catarina, Santa Catarina 88040-970, Florianopolis, Brazil; richard.demo@ufsc.br

**Keywords:** Wireless Sensor Networks, Information Centric, big data, Internet of Things

## Abstract

Wireless Sensors Networks (WSNs) are an essential element of the Internet of Things (IoT), and are the main producers of big data. Collecting a huge amount of data produced by a resource-constrained network is a very difficult task, presenting several challenges. Big data gathering involves not only periodic data sensing, but also the forwarding of queries and commands to the network. Conventional network protocols present unfeasible strategies for large-scale networks and may not be directly applicable to IoT environments. Information-Centric Networking is a revolutionary paradigm that can overcome such big data gathering challenges. In this work, we propose a soft-state information-centric protocol, ICENET (Information Centric protocol for sEnsor NETworks), for big data gathering in large-scale WSNs. ICENET can efficiently propagate user queries in a wireless network by using a soft-state recovery mechanism for lossy links. The scalability of our solution is evaluated in different network scenarios. Results show that the proposed protocol presents approximately 84% less overhead and a higher data delivery rate than the CoAP (Constrained Application Protocol), which is a popular protocol for IoT environments.

## 1. Introduction

Large-scale Wireless Sensor Networks (WSNs) are the main producers of big data, being an essential element of the Internet of Things (IoT) and presenting several applications, such as Smart Cities, Smart Grids, Smart Agriculture, Smart Transportation, Smart Industry, and Environmental Monitoring [1,2,3], as well as other Smart Cyber-Physical Systems [4]. The term smart refers to data collection for information extraction and intelligent decision support, which pushes IoT to one of its main goals: Facilitating human life. Smart Cities can improve the quality of life of citizens in many aspects: Traffic control, air pollution monitoring, safety, and fast emergency response times, among others. In addition, the large amount of data collected by sensor nodes can be used to construct predictive models and to plan specific strategies [5]. Agriculture, for example, can benefit from WSNs to improve productivity, optimize resources (water, energy, fertilizers, pesticides, and so on), control pests, and reduce costs and environmental impact [6,7].

IoT is formed by domains, represented by distinct WSNs [8]. The huge set of data produced by sensor nodes that constitute IoT domains, which is extremely difficult to collect, format, store, manage, analyze, and visualize using the methodologies of traditional computing approaches, is called big data [9].

Big data gathering is a challenging task [10,11] due to the volume, velocity, and variety of data [12]. Velocity refers to the rate of data generation from a wide variety of sources. In the IoT environment, data comes especially from sensors, but also from applications and other devices. The volume refers to the large amount of data generated by WSN, where an energy-efficient protocol is required. Furthermore, the particular characteristics of WSNs also represent a challenge for big data gathering. WSNs are considered Low-Power and Lossy Networks (LLNs): Typically, a multi-hop mesh network consisting of a large number of resource-constrained devices (limited energy, storage, and processing power) that communicate over unreliable links with a low data rate. Instability of the wireless links caused by mobility, depletion of energy resources, failures, and wireless channel effects makes packet loss a common event. Therefore, traditional protocols may not be directly applicable to the constrained IoT environment [13].

Currently, data monitoring is a key activity in WSNs. However, the ability to send commands/ queries from a central unit to the network nodes is increasingly becoming fundamental [14]. Conventional network protocols rely on message flooding or demand to send one command/query to every destiny node. These strategies are unfeasible for large-scale networks. On the other hand, Information-Centric Networking (ICN) is a promising paradigm for the Future Internet architecture, which can help overcome some challenges related to big data gathering.

ICN represents a revolutionary approach in the network field [15,16]. The basic idea is to enable data routing based on the information provided by nodes (content), rather than based on the address of the nodes. Data routing based on content is scalable, and facilitates data retrieval as well as application development, since data is requested by name and its specific physical location is transparent.

The potential and benefits of the ICN paradigm in WSNs have been extensively investigated. In [17,18,19], the authors proposed the use of elements of cognition in information-centric wireless networks to dynamically identify good data delivery paths, extend network lifetimes, improve reliability, and minimize latency. In [20], a distributed algorithm was proposed to provide information-centric data collection to mobile devices. The potentialities of the ICN paradigm applied to WSNs were identified in [21]. The authors also proposed some enhancements for reducing traffic overhead and collisions. Problems created by IP addressing style were discussed in [22]. In [23], the authors presented an scheme to apply the ICN paradigm in industrial environments, in order to reduce the bandwidth overhead caused by periodic traffic.

There are a number of developing projects around the world that investigate different ICN approaches [13,24]. A particular case, Content-Centric Networking (CCN), proposed in [25], has gained the attention of researchers for its applicability in WSNs [13,16,21,26,27,28]. However, CCN was conceived for the internet, and requires appropriate modifications to be applied to WSNs.

In this paper, we propose ICENET—Information Centric protocol for sEnsor NETworks. ICENET is a soft-state information-centric protocol for big data gathering in large-scale WSNs, inspired by the CCN approach. The proposed protocol enables content-based routing over a tree-based topology. Data routing based on content allows clients to interact semantically with the network to request data. In this context, a user can request data from the network using SQL-based queries which are processed in-network by the protocol. A single message collects data from multiple nodes during a certain period of time. The tree-based structure is constructed by the Routing Protocol for Low-Power and Lossy Networks (RPL) [29], introduced by the Internet Engineering Task Force (IETF) as the standard routing protocol for LLNs. Due to its hierarchical structure, tree-based routing is a potential candidate for optimizing bandwidth usage. As the routing structure is hierarchical, parent nodes send messages only to children and vice versa, allowing the number of messages to be reduced. Many studies have used tree-based routing to improve bandwidth usage and save energy in large-scale multihop WSNs [30,31,32]. In a ICN context, parent nodes can forward queries based on the content provided by their child nodes. This strategy also significantly reduces the number of message transmissions. A soft-state mechanism makes the protocol robust and prepared for packet losses and changes in the network topology: Messages used to request data and to keep network states alive are periodically refreshed, according to timers that adapt to network stability. The goal is to send few messages when the network is stable, in order to save energy, and send messages aggressively during periods of instability, enabling a quick reaction to topological changes. Simulation results show that ICENET is suitable for big data gathering in large-scale WSNs. The proposed protocol is scalable, energy-efficient, suitable for collecting large volumes of data, and presents a low overhead. Compared to the Constrained Application Protocol (CoAP) [33], ICENET presents a higher success rate and up to 84% less overhead.

The rest of this paper is organized as follows. In Section 2, we investigate related works proposed in the literature. In Section 3, the ICN paradigm and CCN approach are explained and the fundamentals of RPL are presented. ICENET is presented in Section 4. Simulations results are given in Section 5. Finally, Section 6 concludes the paper and outlines future research directions.

## 2. Related Works

Protocols for IoT environments can be classified into two groups: Address-centric and information- centric. Address-centric protocols use the address of nodes for data routing, while information-centric protocols use the information provided by the nodes for routing. The main protocols representing the two groups are discussed in the next subsections and their features are compared in Table 1.

### 2.1. Information-Centric Protocols

The Directed Diffusion (DD) protocol [34] is a classical routing solution for sensor networks, while Van Jacobson et al. [25], later, suggested the initial ideas that led to the design of the first information-centric routing protocol for WSNs. Recently, the search for alternative architectures for the Future Internet and the increasing number of IoT applications has once again drawn attention to information-centric approaches, and a number of works investigated the applicability and potential of the paradigm in WSNs [13,16,17,21,22,26,27,37].

Although being a classic solution, DD relies on flooding which incurs in considerable overhead and is not suitable for large-scale wireless networks. To request information, users insert queries (called ‘interests’) into the network, at some arbitrary sink node. A single interest message retrieves data from multiple nodes. Initially, the sink broadcasts the received interest at a low data rate. This interest message is exploratory and aims to determine if there are nodes to respond to the received query. Sensor nodes also broadcast received interests, such that interest messages are flooded over the whole network. Then, if a node is a data source for an interest, it forwards a data message to each neighbor node from which the interest was received. Thus, data messages return along the reverse path of interest message propagation, so that the sink eventually receives data from multiple paths. DD adopts a mechanism called reinforcement to ensure the use of optimal routes between the sensors and the sink, and also to perform route repair. Then, after receiving data messages from multiple paths, the sink node selects one neighbor and re-sends the interest at higher data rate, according to local rules. One example of a local rule is to reinforce any node that transmits data faster than others. In order to monitor the quality of the paths and to guarantee data delivery, the sink continues to receive data from multiple paths. However, the selected path sends data at a higher rate. Furthermore, DD implements a simple caching mechanism, which is, nevertheless, not sufficient to minimize the damage of constantly flooding a resource-constrained network in both up and down directions.

CCN-WSN has been proposed, in [28], as a variant of the CCNx protocol designed for PCs. Since CCNx is not directly applicable to WSNs, CCN-WSN redesigns the message format to cope with the IEEE 802.15.4 standard [38], as well as modifying the naming strategy, according to the information provided by the sensors. Then, CCN-WSN is employed straight away on top of IEEE 802.15.4, avoiding upper layer protocols in order to reduce overhead. However, CCN-WSN still has a high overhead since it relies on flooding; while nodes need to maintain a table, listing every user request. With CCN-WSN, nodes broadcast an interest message to request content. If the receiver node has the requested content, it broadcasts a content object. Data is only sent in response to an interest and consumes the interest message. Therefore, periodic data sending (‘convergecast’) is not provided. Moreover, as in DD, data return along the reverse path of the interest propagation and a simple caching mechanism is adopted. Consequently, CCN-WSN still inherits some features that make it less appropriate for WSNs.

Recently, the Content Centric Routing (CCR) protocol [35] was proposed for efficient in-network data aggregation and retrieval in IoT networks. The authors developed an objective function for the RPL protocol that enabled routing based on content, such that data messages with different content types were forwarded through different routes. To this end, the nodes execute the objective function in order to select the relay node for each traffic content. First, the node broadcasts a message informing the outgoing traffic content types, the corresponding traffic volume, and a candidate selection criteria. Candidate nodes respond with a message containing their node id and their estimated lifetime, if they are chosen to forward the data. Then, the source node ranks the candidates using the proposed objective function, and assigns a relay node to the corresponding traffic. Thus, nodes maintain a different routing entry for each piece of content. Moreover, received data are locally processed and aggregated. As a consequence, the CCR protocol has several advantages: Avoiding transmission of redundant network traffic, providing energy efficiency, and prolonging the network lifetime. However, only multi-point to point data collection scenarios are considered by [35], while the forwarding of data requests to data sources was not investigated.

### 2.2. Address-Centric Protocols

Several address-centric protocols have been proposed for IoT environments. Two of the more popular protocols are the Message Queue Telemetry Transport (MQTT) [36] and the Constrained Application Protocol (CoAP) [39]. Both MQTT and CoAP are application layer protocols, whose major difference is that the former uses TCP as a transport protocol while the latter runs on UDP [40]. The reason for designing an UDP-based application layer protocol is to avoid all TCP connection overheads for connection establishment and closing, which makes the protocol more suitable for IoT environments. Compared to other application layer protocols, CoAP presents the lowest overhead and has received widespread acceptance [41,42].

MQTT uses a topic-based publish-subscribe architecture. Intelligent devices (publishers) send messages to an address called a topic in a server (broker). Clients register as subscribers for certain topics and the broker informs then when publishers update topics of interest. Publishers are data sources that transmit information to subscribers through the broker. The reliability mechanism offers three Quality of Service (QoS) levels for message delivery. Although MQTT is designed to present a low overhead, publishers constantly send data to the broker, even when there are no interested subscribers. This mode of operation is not efficient and causes unnecessary consumption of network resources.

CoAP is based on Representational State Transfer (REST) and was developed to operate with HTTP. REST is an architectural style that makes information available as resources identified by Universal Resource Identifiers (URIs). Two architectures are supported: Request-response and resource-observe. If the request-response architecture is adopted, clients send requests to a server and receive data in response to these requests. The observe architecture allows a client (observer) to register its interest in a certain resource indicated by a URI. When the IoT device updates an URI with a new value, all observers are notified. Since UDP does not provide reliable message delivery, CoAP implements its own reliability mechanism. Messages are either confirmable or non-confirmable. Confirmable messages require an acknowledgment from the receiver node. CoAP also supports multicast messages to a group of devices [43]. However, this mode of communication presents important limitations [44]. First, multicast messages are not confirmable, and therefore no reliability mechanism is provided. In addition, multicast messages prevent the adoption of a caching mechanism. Another important drawback refers to scalability. Considering large-scale WSNs, the number of possible multicast groups may not be feasible, given the available resources. Furthermore, the cost of maintaining a multicast forwarding topology is prohibitively expensive and demands the adoption of special multicast protocols [45]. Due to the unfeasibility of multicast communication in large-scale wireless networks, a client interested in information from various data sources should address a request for each of them. Each node corresponds to a distinct URI. This means that the server must know the information provided by each of the nodes. This centralized approach generates high overhead communication to keep information about the nodes up to date. In addition, sending multiple requests to the network to get the same information is not efficient.

Regarding WSNs, an energy-efficient protocol is required in order to gather the huge amount of data produced by the large number of sensors, but the strategies adopted by traditional protocols are not suitable. Flooding, forwarding a query/command to each destination node, or periodically sending data to a central device causes interference, delays, and a reduced network lifetime. In addition, centralized solutions must be avoided because of the unacceptable communication overheads involved in network topology discovery. Moreover, none of the mentioned protocols offer a reliability mechanism that considers both the highly dynamic environment of wireless communications and the scarcity of computational and energy resources of sensor nodes.

## 3. Fundamental Concepts

In this section, we present some fundamental concepts employed by ICENET, which will be described in detail in Section 4.

### 3.1. ICN Paradigm and CCN Approach

**Figure 1:**
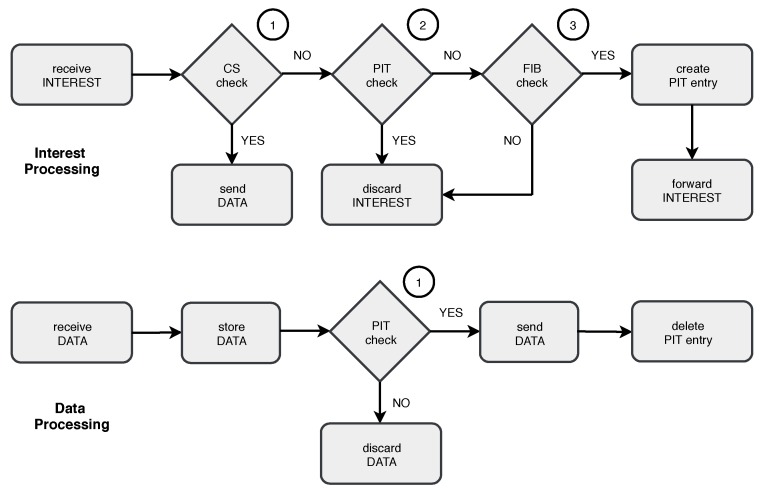
Message processing and forwarding in Content-Centric Networking (CCN).

Every CCN node presents three data structures: (i) A FIB (Forwarding Information Base), which is similar to a routing table and is used to forward data requests to data sources; (ii) a PIT (Pending Interest Table) to forward incoming data packets to the requesters; and (iii) a CS (Content Store) for content caching. Each piece of content are identified by a name, which is persistent, unique, and hierarchical. Figure 1 shows the CCN message engine.

There are two CCN messages: ‘Interest’ and ‘data’. Interest messages are broadcast over the network to retrieve information. If a node receives an interest message and stores the solicited information, it replies with a data message. As CCN was conceived for the internet, interest and data messages have a one-to-one relationship: Nodes transmit a data message only in response to an interest, consuming the interest. However, this mode of operation is not energy efficient and, besides, users may be interested in data provided by multiple nodes in the WSN. If a node is unable to answer an interest, the PIT is checked. A matching PIT entry means that the node has already received the same interest from another user or the message is duplicated, and so the interest is discarded. Finally, if there is no PIT entry, the longest prefix matching is used to select a FIB entry to forward the received interest to a potential data source. After the FIB entry is selected, a new PIT entry is created. Incoming data packets are stored in the CS and then forwarded to the requesters, according to the PIT entries. Big data gathering can create a massive number of interests. Consequently, the PIT may overflow and the network may collapse [15].

The information requested in WSNs can be a set of data provided by a large number of nodes and CCN does not allow data collection from multiple nodes. Besides, CCN routers have to maintain tables, listing every user request they forward and next hops to content names, which incurs a considerable storage overhead. Moreover, users must express interest for each data packet. This mode of operation is unsuitable for constrained environments, and does not support alerts and emergency notifications.

### 3.2. RPL Basics

RPL presents an energy-efficient solution to construct and maintain a tree topology with redundant paths, which reacts quickly to connectivity changes, called a Destination Oriented Directed Acyclic Graph (DODAG) [29]. The network topology may present multiple DODAGs, rooted at different gateway nodes. By default, each sensor node has a preferred parent node to forward data packets toward the root, and multiple alternative parents to be used as backup routes [14]. A DODAG can support three traffic patterns: (i) Upstream traffic from nodes toward the gateway (multipoint-to-point); (ii) downstream traffic from the gateway toward nodes (point-to-multipoint); and (iii) traffic between nodes (point-to-point).

**Figure 2:**
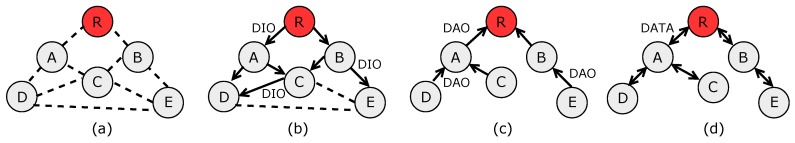
(**a**) A general wireless network. (**b**) Downward route construction. (**c**) Upward route construction. (**d**) Data traffic.

There are three RPL messages: DIO (DODAG Information Object), DIS (DODAG Information Solicitation), and DAO (Destination Advertisement Object). Figure 2 shows DODAG construction, which starts when the root sends a multicast DIO message, containing configuration parameters and routing metrics (rank). This information is used by nodes to evaluate the path cost and select the preferred and alternative parents. Receiver nodes compute their own rank and then send the DIO. Nodes send DIS messages to request DIOs from the neighborhood, in order to join DODAG or update routing information. DAO messages are used to establish downward routes. In addition, RPL supports two modes of downward traffic: Storing or non-storing. In the storing mode, nodes store source-routing table entries for destinations learned from DAOs. When a data packet is received, the node examines the routing table to decide the next hop. Conversely, in the non-storing mode, only the root stores a downward routing table and data packets are source routed to the destination.

Even after DODAG construction, nodes keep transmitting DIO messages in order to maintain the routing topology. The Trickle Algorithm [46] is run by RPL nodes to determine the rate for sending DIOs. RPL is highly efficient, in terms of energy and message overhead, for maintaining a tree-based wireless topology. Additionally, it can rapidly adapt to changes in channel conditions and network topology. Given these features, RPL is assumed as the underlying protocol for our proposed information-centric architecture.

## 4. ICENET—Information Centric Protocol for sEnsor NETworks

**Figure 3:**
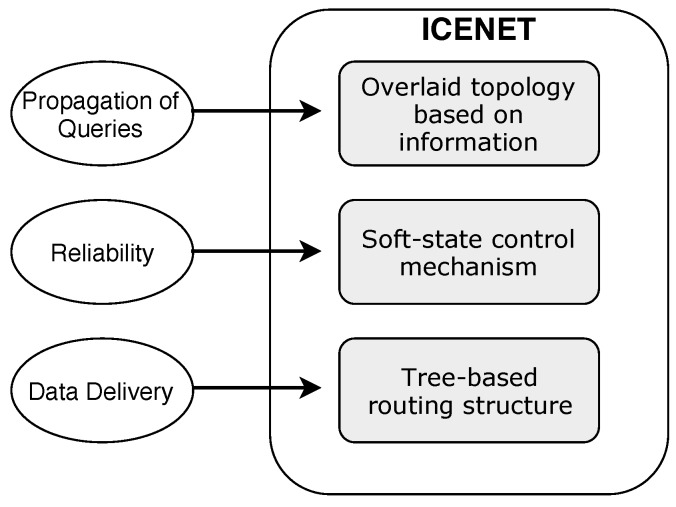
Information Centric protocol for sEnsor NETworks (ICENET) components.

ICENET is composed of three distinct elements, which operate independently: (i) A tree-based routing structure supported by RPL, for data message delivery; (ii) an overlaid routing topology based on information, for propagation of queries (interest messages); and (iii) a soft-state control mechanism, for reliability. Figure 3 shows the task attributed to each ICENET component.

We assume that queries are injected into the network by a gateway node (root), while the sensor nodes send data only in response to these queries. The tree-based structure constructed by RPL is in charge of delivering data messages to the gateway, while the overlaid routing topology, based on information, is in charge of forwarding queries to data sources.

Data messages are relayed from child nodes to the parent node until they reach the gateway. Queries are forwarded from parent nodes only to child nodes, based on the information provided by them. ICENET nodes work collaboratively, in a distributed approach, to deliver queries to potential data sources. Centralized approaches are infeasible in large-scale deployments, since global network information is often required. In dynamic environments, centralized approaches are even more harmful, because they introduce delays and communication overhead to maintain the network information updates.

In order to prevent flooding, which must be avoided in large-scale IoT deployments [37], the parent node forwards a query only if the child nodes have routes to the data sources. For this, the nodes maintain a forwarding table (called the FIB) which stores the information provided by their child nodes. In comparison to other approaches, the size of this table is significantly reduced as it does not store routes for the entire network. If we apply a conventional approach in large-scale networks, the size of the tables maintained by the nodes may overflow and lead to network collapse. Another advantage of hierarchical structures, such as trees, is to avoid the use of a frequent route discovery mechanism. In addition, trees are the most appropriate structure for data aggregation and periodic data transmission [30].

**Figure 4:**
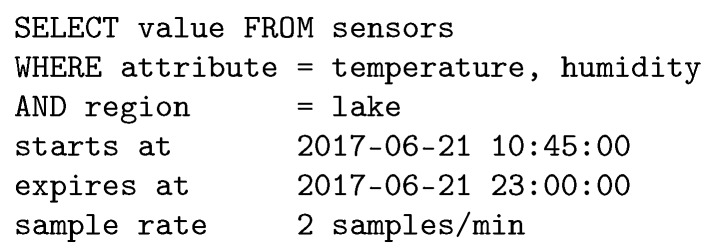
Query example to extract information from an environmental wireless sensor network (EWSN).

The information-centric paradigm allows clients to retrieve data without the knowledge of the addresses of the nodes. As in [47], we assume that information is extracted from the network using a query language. Consider, for instance, an environmental WSN (EWSN) covering a large monitoring area containing a lake. A query example, intended to collect information about temperature and humidity in the lake, is shown in Figure 4.

Nodes decide whether to forward a received interest message, according to the information stored locally in the FIB, whose entries are composed of attribute (ATTR), reachable region (REGION), and data sources (DS):

FIBEntry={ATTR,REGION,DS}.

The ATTR field refers to some information (physical monitored parameter), the REGION refers to a specific location, and DS represents the set of child nodes that provide the information. Only interest messages received from the parent node are processed. Interest messages received from nodes other than parents are discarded.

Nodes store a received interest in a local table, called the Icache (Interest Cache), until it expires. Icache entries are composed by identification (ID), timestamp (TS), attribute (ATTR), REGION, expiration time (ET), and data sample rate (SR):

IcacheEntry={ID,TS,ATTR,REGION,ET,SR}.

The ID field refers to an identification assigned to the interest by the gateway and TS determines a specific time point to start the data collection.

If a node receives an interest message and possesses the requested data, it sends a data message to the parent node. Data caching is performed only at the gateway node. Since the network topology is based on a tree, interests are first received at the root (gateway) and data always converges to the root. Therefore, data caching performed by the gateway reduces the number of messages transmitted trough the network.

In order to make ICENET robust and suitable to a dynamic environment, we propose a soft-state based design. Soft-state protocols use periodic refresh messages to keep the network state updated. State refers to the information stored in the network nodes. Soft-state protocols adapt faster and present great robustness to changes in network conditions [48]. For this reason, ICENET periodically refreshes interests stored in Icaches and the information stored in FIBs. ICENET incorporates Trickle Algorithm [46] principles to determine the rate at which to refresh interests, which will be further detailed in Section 4.1. Trickle timers adapt dynamically to network conditions, so that the message rate increases during periods of instability, and decreases otherwise.

**Figure 5:**
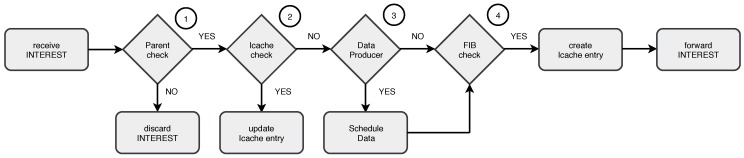
Interest message processing and forwarding in ICENET.

Figure 5 shows interest processing and forwarding in ICENET. Interests are processed by nodes in-network, improving scalability. In addition, a single message can collect data from multiple nodes and for a certain time interval.

Sensor nodes only process interests received from the parent node (denoted as Condition 1 in Figure 5). Then, the receiver node checks the Icache (Condition 2). A matching interest ID means that the interest was already received, so that the node only updates the entry if new information is provided. Otherwise, the node checks if it can provide the requested data (Condition 3) and schedules the data collection. Then, the node checks the FIB (Condition 4) in order to forward the interest to data sources. If data sources are found, the node creates an Icache entry, broadcasts the interest, and starts the execution of the Trickle Algorithm. Trickle allows interest messages to be delivered more efficiently and with less overhead, in situations where communication links are unstable or when the topology changes.

### 4.1. Trickle Algorithm for Interests

The Trickle Algorithm is a scalable, powerful, and simple mechanism that can be applied to a wide range of protocol design problems [49]. ICENET adopts Trickle principles to efficiently refresh the interests stored in the node Icache. This is accomplished by defining specific consistency and inconsistency events. Consistent messages, or events, do not change the perception of the node about the network topology or channel conditions, and therefore reflect stability. ICENET nodes consider that to receive a data message from one of the child nodes represents a consistency, while not receiving any data message from any of the child nodes represents an inconsistency.

The Trickle Algorithm presents three configuration parameters: Minimum interval size ($I_{\min}$), maximum interval size ($I_{\max}$), and a positive integer constant (*k*). In addition to these parameters, there are three variables: Current interval size (*I*), a random time point (*t*) in the interval [I/2,I], and a consistency counter (*c*). If a consistent message/event is received or detected, *c* is incremented. At time *t*, ICENET nodes broadcast the stored interest if c<k. When *I* expires, the time interval is doubled, in order to slow the communication rate exponentially. If an inconsistency is detected, Trickle resets the interval length *I* to the initial value ($I=I_{\min}$), in order to increase the sending rate, so that the nodes quickly adapt to changes in network conditions. Whenever *I* is reset, Trickle sets *t* to the interval [I/2,I]. Table 2 shows the main operations of the Trickle Algorithm.

ICENET sets the parameter $I_{\min}=\mathrm{SP}$ (sample period) of the interest. The intuition is that, after sending an interest message, the node would start receiving data packets after a time approximately equal to the sample period. This assumption assumes ideal channel conditions, which means error-free links and no losses in the MAC layer. However, in real environments, the interest message may not be received by all child nodes. For this reason, ICENET does not require all child nodes to answer an interest. This can be parametrized by the constant *k*, which represents the number of child nodes that should answer the interests. This is a very important feature, as we can tune the protocol behavior depending on the application requirements.

### 4.2. Naming Strategy

In the Information-Centric paradigm, the element “Name” identifies the information provided by a certain node and, therefore, plays a key role in communication. In ICENET, naming has two elements: Node attribute (ATTR) and node region (REGION). These elements describe the physical parameter monitored by the node and location of the node, respectively. The node attribute is an alphanumeric flat name and the node region is a hierarchical name structure, composed of one or more alphanumeric components separated by “\”. The deployment region is successively divided into subregions, each represented by a different component name. The hierarchy of component names are application-dependent and must be defined before the WSN deployment.

**Figure 6:**
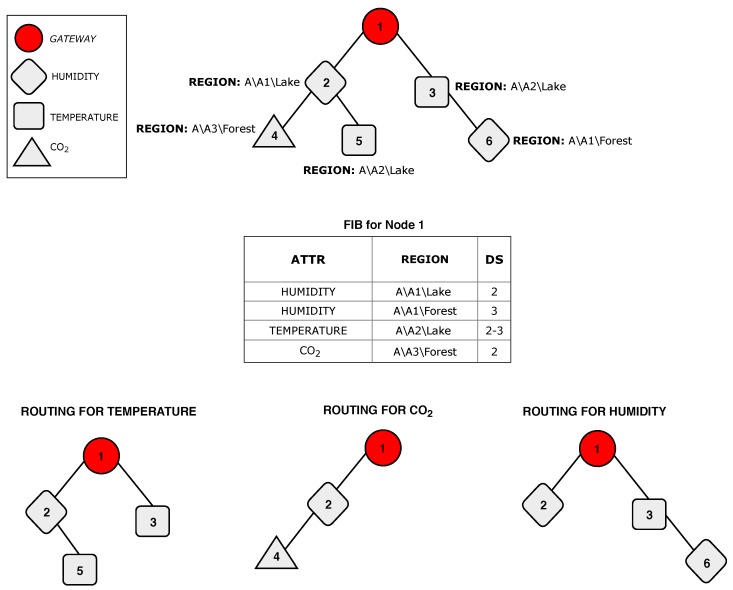
Examples of content-based routing over a tree structure.

**Figure 7:**
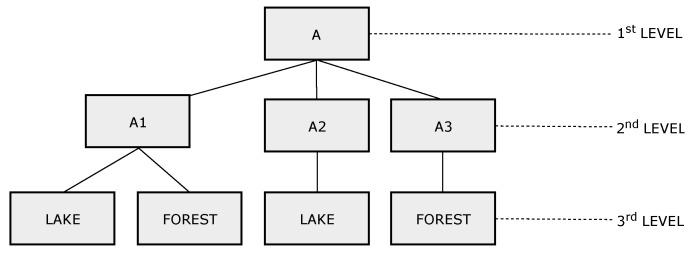
Name tree-hierarchy of component names.

Figure 6 shows a tree-based network topology example, indicating the ATTR and REGION elements in each node. For example, node 2 monitors the parameter humidity, and the node region has three levels: Region A, subregion A1, and subregion Lake. A node sends data in response to an interest whenever the REGION field of the message partially matches the node region. Therefore, the field REGION of the interest message defines the scope of the queries. Considering the network shown in Figure 6, if a client asks for the parameter humidity in the subregion A1 (A\A1), nodes 2 and 6 will send data in response to the received interest. Otherwise, if the client asks for humidity in the lake located at subregion A1 (A\A1\Lake), only node 2 processes the interest and sends data to the parent node. Figure 7 shows the name tree (hierarchy of component names) for the network topology shown in Figure 6.

Names are not unique, because multiple sensor nodes sensing the same physical parameter can be deployed in the same region. Additionally, to fit into the IEEE 802.15.4 standard, the maximum length of a content name is 50 octets and is limited to five components with a maximum of 15 octets each. A possible solution to overcome this limitation is to translate component names into binary code.

### 4.3. Procedure for FIB Population and Update

Update messages are used to populate and update FIB entries. When the execution of ICENET starts, all sensor nodes unicast an update message to the parent node containing all distinct regions currently reachable by the node and the respective attributes. Figure 6 shows an example of the FIB at the gateway node. FIBs allow the use of different routes for each piece of content, as shown in Figure 6. The construction of different routes reduces the network traffic, extends the network lifetime, and improves the scalability and efficient use of the wireless channel.

A local timer, denoted by $T_{u}$, and a configuration parameter n>0 are used by the nodes to set the rate for transmitting update messages. When $T_{u}$ expires, the node checks for topological changes. If the parent node has changed, or if the FIB has been updated by adding or removing regions or attributes, the node unicasts an update message, to inform the parent node. Nodes must also send an update message after $n\cdot T_{u}$ because FIB entries expire after this period, to avoid sending interests to invalid routes.

### 4.4. ICENET Architecture

**Figure 8:**
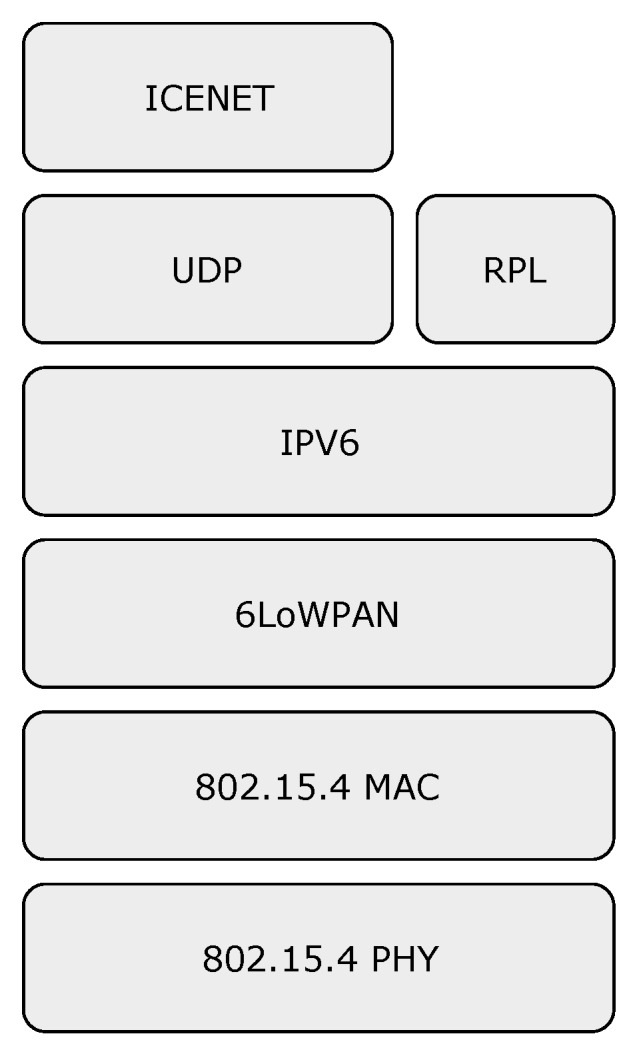
The ICENET protocol stack.

The ICENET protocol stack is shown in Figure 8. Communication at the Physical (PHY) and Medium Access Control (MAC) layers are supported by IEEE 802.15.4 [38], which is the standard protocol for communication at lower layers in IoT architectures. The 6LoWPAN adaptation layer [50] allows the transmission of IPv6 packets over IEEE 802.15.4, whose most pertinent functionalities are compression, fragmentation, and reassembly of IPv6 packets. The reason for choosing UDP as transport protocol is to avoid the overhead and complexities of TCP. Moreover, since ICENET has its own reliability mechanism suitable for WSNs, the reliability functions of TCP are not necessary.

## 5. Simulations and Results

In order to evaluate its efficiency and scalability, ICENET is compared to CoAP, which was chosen as it represents one of the most popular protocols for IoT environments [51,52]. From this point forward, we assume that the term “query” represents an information request for both evaluated protocols.

### 5.1. Network Modeling and Simulation Environment

The following assumptions are made about the network:

1. Links between nodes are symmetric: For each link i⇒j, there is a reverse link j⇒i.
2. The gateway is located at the center of the topology.
3. Sensor nodes are equal in terms of processing power, radio technology, battery, and memory.
4. The gateway node has unlimited energy and higher storage and processing capacity.
5. The CSMA/CA protocol is assumed for medium access control.

Assumptions 1 and 4 are usual in WSN simulations, and aim to simplify the interpretation of the results. Assumption 2 does not affect the comparison of protocols. We place the gateway at the center of the topology because the distribution of data messages in the network becomes more uniform as the number of nodes close to the gateway increases. Assumption 3 is also usual for WSN simulations, because the gateway commonly has essentially unlimited energy and a higher storage and processing capacity in real scenarios. Assumption 5 refers to the use of IEEE 802.15.4 for medium access control.

We consider grid network topologies with a random disturbance in the position of nodes. To perform the simulations, we use the software Mathematica [53], along with the SensorSim library [54]. The SensorSim library uses the transmission parameters of the IEEE 802.15.4 [38]. Table 3 shows the Trickle parameters chosen to control the forwarding rate of interest messages, as well as the parameters $T_{u}$ and *n* used to control the forwarding rate of update messages. The value assigned to $I_{\max}$ describes the number of doublings of the minimum interval size ($I_{\min}$).

### 5.2. Wireless Channel Model

We assume a wireless channel subject to quasi-static fading. The instantaneous signal-to-noise ratio (SNR), measured by a node *j* from a packet received from a node *i*, is given by [55]:

(1) $$\gamma_{ij}={\left|h_{ij}\right|}^{2}\;\frac{P_{r}}{N},$$

where $P_{r}$ is the average received power, *N* is the noise power, and $h_{ij}$ is the channel gain. The channel fading $h_{ij}$ follows the Nakagami-*m* distribution, while $|h_{ij}{|}^{2}$ follows the Gamma distribution. The probability density function of $\gamma_{ij}$ is, thus,

(2) $$f_{\gamma_{ij}}\left(x\right)=\frac{{\left(m/{\bar{\gamma }}_{ij}\right)}^{m}\;x^{m-1}}{\Gamma \left(m\right)\;e^{mx/{\bar{\gamma }}_{ij}}},$$

with $\Gamma \left(a\right)=\int_{0}^{\infty }y^{a-1}\;e^{-y}dy$ being the Gamma function and ${\bar{\gamma }}_{ij}=P_{r}/N$. The average received power is given by

(3) $$P_{r}\left(d_{ij}\right)\;\left[\mathrm{dBm}\right]=P_{r}\left(d_{0}\right)\;\left[\mathrm{dBm}\right]-10\;\alpha \log_{10}\left(\frac{d_{ij}}{d_{0}}\right)+S,$$

where $d_{ij}$ is the distance between nodes *i* and *j*, $P_{r}\left(d_{0}\right)$ represents the power received at a reference distance $d_{0}$, the parameter α is the path loss exponent, and *S* represents the shadowing, which follows a zero-mean Gaussian distribution with variance $\sigma^{2}$ in dB [56]. We assume a homogeneous environment, with the path loss exponent α=3, Nakagami-*m* fading with parameter m=2, and shadowing variance $\sigma^{2}=5$ dB, for all nodes.

### 5.3. Simulation Setup

To run the simulations we considered randomly-generated topologies with the number of nodes *N* ∈{100,200,300}. For each *N*, 18 simulations, divided into three groups of six rounds, were performed. Each simulation group assumed a different sampling rate SR∈{5,10,30} s. The deployment region *R* presented three levels: {A, A\A1, A\A1\A1.1}. During a simulation round, one single query asking for one physical parameter for 300 s, was injected in the network. The same injected query was processed by both ICENET and CoAP protocols. Four physical parameters were monitored by the network, but each node monitored only one parameter. Inside the simulation groups, the injected queries were evenly distributed among the three *R* levels.

### 5.4. Results

**Figure 9:**
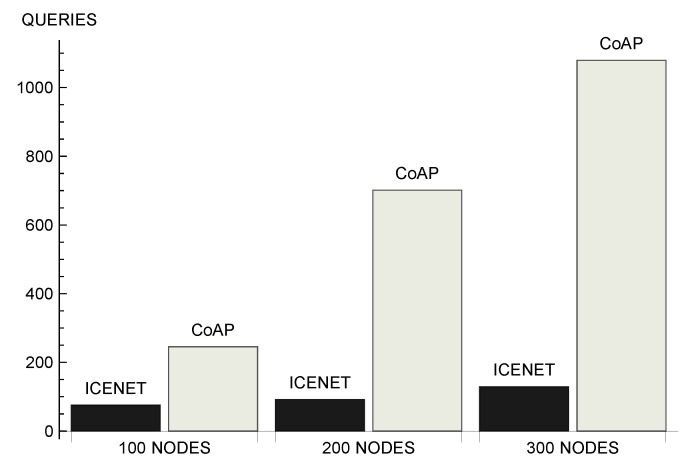
Number of queries forwarded by nodes.

Figure 9 shows the average number of queries forwarded by nodes per simulation round. This is an important metric, because it impacts directly on network lifetime—data communication is the most energy-consuming activity of the sensor nodes [52,57]. In addition, the number of messages is related to the efficient use of the wireless channel, interference, and delay. We can observe that, the larger the number of nodes, the more significant was the difference between the number of queries forwarded by the two protocols. In a scenario with 100 nodes, ICENET nodes forwarded 75.60 queries on average, while CoAP nodes forwarded 245.44 messages on average. In a scenario with 300 nodes, ICENET nodes forwarded 129.33 queries, while CoAP nodes forwarded 1079.61 messages. Comparing the two scenarios, ICENET increased the number of forwarded queries by 71% when the number of nodes increased from 100 to 300, while CoAP presented an increase of approximately 340%. In other words, in a scenario with 300 nodes, CoAP forwarded approximately 8 times more queries than ICENET. This difference occured because CoAP unicasted a request to each node that monitored the physical parameter. In addition to the largest number of queries initially generated by each node, the unicast communication pattern caused the retransmission of lost messages by the MAC layer.

**Figure 10:**
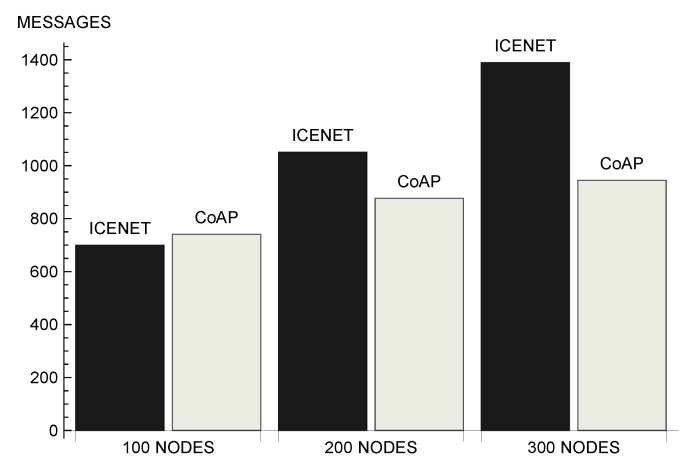
Number of data messages received by gateway.

Figure 10 shows the average number of data messages received by the gateway node for each simulation round. Although CoAP nodes forwarded more queries, in scenarios with 200 and 300 nodes the ICENET gateway received a larger number of data messages. In a scenario with 100 nodes, the CoAP server received approximately 6% more data messages than the ICENET gateway. However, in a scenario with 300 nodes, the ICENET gateway receiveD approximately 47% more data messages. When *N* is large, ICENET presented the best results, because the large number of query messages sent by CoAP congested the network. Network congestion is related to delay, interference, and data loss. The results presented by the scenario with 100 nodes shows that the strategy adopted by CoAP to forward queries performed better for networks consisting of a few nodes. The number of messages generated to unicast a query to each node that monitored the requested physical parameter, and the subsequent retransmissions performed by the MAC layer, were not sufficient to congest the network and, therefore, CoAP delivered a larger number of data messages.

**Figure 11:**
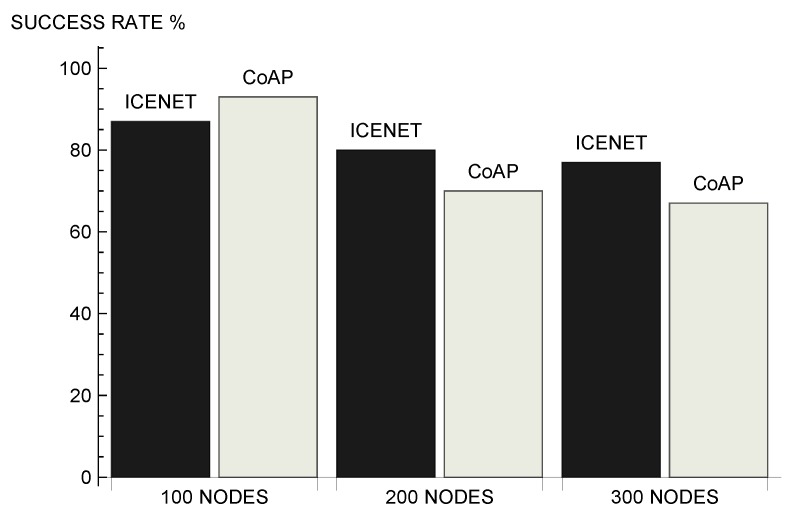
Average success rate.

Figure 11 shows the average success rate presented by the evaluated protocols. Success rate is the relationship between the theoretical number of data messages and the data messages actually received. The theoretical number of data messages corresponds to the number of messages that would be received in an ideal scenario, without packet losses due to the wireless channel impairments, interferences, or multiple accesses. The success rate is related to the number of data messages received by the gateway. In a scenario with 100 nodes, CoAP presented a success rate 5% higher than the success rate presented by ICENET. However, in scenarios with 200 and 300 nodes, ICENET presented the best results. In the scenario with 300 nodes, ICENET presented a success rate 9% higher.

**Figure 12:**
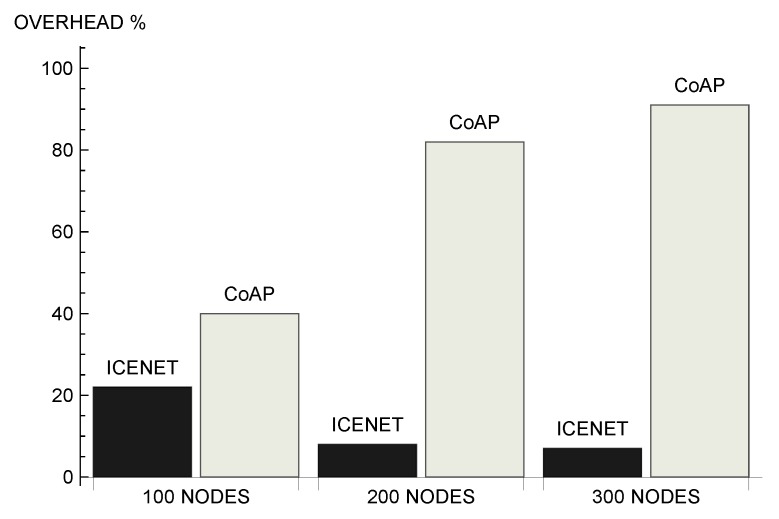
Average overhead.

Figure 12 presents the average overhead associated with the evaluated protocols. Overhead refers to the relationship between the number of forwarded queries and the number of received data messages. ICENET demonstrated itself to be a scalable solution, as it reduced the overhead as the number of nodes increased. This occurred because broadcast queries, taking into account the nodes that can provide the information, became more efficient as the number of nodes able to answer the query increased. On the contrary, the strategy adopted by CoAP led to a significant increase of overhead as the network became larger. Comparing the scenarios with 100 and 300 nodes, ICENET reduced the average overhead in 15%, while CoAP increased the overhead by approximately 52%. In the scenario with 300 nodes, ICENET presented an average overhead of 7%, while CoAP presented an average overhead of 91%.

## 6. Conclusions

In this paper we propose ICENET—Information Centric protocol for sEnsor NETworks, capable of dealing with big data in WSNs. Big data gathering enables several emerging IoT applications, but presents challenges involving huge volumes of data, resource-constrained devices, and network instability. Current IoT applications require not only periodic data sending, but also the forwarding of queries and commands to nodes. The proposed protocol is a more efficient and suitable solution than protocols adopting traditional approaches, due to several factors. Compared to CoAP, a popular protocol designed to IoT environments, the proposed solution presents significantly less overhead during the query propagation phase, while collecting a larger amount of data. Moreover, our solution is more scalable, since the overhead reduces as the number of nodes increases. In addition, the ICN approach allows users to interact dynamically with the network. Lastly, an efficient soft-state mechanism makes the protocol robust to dynamic environments. This is an important feature, since message loss is a common event in wireless networks and sensor nodes are prone to failures. For the continuation of this work, we intend to propose an aggregation strategy to send requested data to clients and to determine the ideal values of the configurable parameters of the proposed protocol.

## Figures and Tables

**Table 1:** Protocol comparison.

PROTOCOL		DD [34]	CCN-WSN [28]	CCR [35]	COAP [33]	MQTT [36]
**Approach**	information-centric	X	X	X		
	address-centric				X	X
**Transport**	UDP			X	X	
	TCP					X
	Specific	X	X			
**Routing**	Flooding	X	X			
	RPL			X	X	X
**Architecture**	request-response	X	X	X	X	
	publish-subscribe					X
	resource-observe				X	
**Reliability**	multiple paths	X				
	not implemented		X			
	confirmable messages			X	X	
	QoS levels					X
**Data from Multiple Nodes**	yes	X	X			
	no			X	X	X

**Table 2:** Operations of the Trickle Algorithm [49].

Event	Action
*I* Expires	Set I=2·I, where $I\leq I_{\max}$
	Set c=0 and randomly pick t∈[I/2,I]
*t* Expires	If c<k then transmit
Consistency Detected	c=c+1
Inconsistency Detected	Set $I=I_{\min}$ and randomly pick t∈[I/2,I]

**Table 3:** Parameters for refreshing interests and updating FIBs.

Parameter	Description
$I_{\min}$	SP (sample period) of the Interest
$I_{\max}$	4
*k*	∑ child data sources (|DS|)
consistency	Receive a Data message from a child node
inconsistency	Do not receive any Data message from any of the child nodes
$T_{u}$	1 s
*n*	0.004 s
